# Should more patients be offered repair for mitral valve endocarditis? a single-centre 15-year experience

**DOI:** 10.1186/s13019-022-01997-2

**Published:** 2022-09-30

**Authors:** Clarissa Ng Yin Ling, David Bleetman, Soumik Pal, Hing Chi Kristie Leung, Habib Khan, Donald Whitaker, Olaf Wendler, Ranjit Deshpande, Max Baghai

**Affiliations:** 1 Department of Cardiothoracic Surgery, King’s College Hospital, London, UK; 2 Department of Surgery and Cancer, Imperial College London, South Kensington Campus, London, SW7 2AZ UK; 3 University College London, London, UK

**Keywords:** Endocarditis, Mitral valve, Mitral valve repair, Mitral valve replacement

## Abstract

**Objective:**

To describe the long-term outcomes of mitral valve repair (MVr) versus mitral valve replacement (MVR) in patients with native valve infective endocarditis (IE) at a centre with high-repair rates.

**Methods:**

We conducted a retrospective single-centre cohort study. From 2005 to 2021, 183 patients with active or healed native valve IE were included. The primary outcome was long-term mortality. Patient status was last confirmed 31 March 2021. Secondary outcomes were post-operative MR, MV reoperation, length of post-operative intensive care stay and total hospital stay.

**Results:**

85 patients (46.4%) underwent MVr and 98 (53.6%) underwent MVR. Follow-up was 98.9% complete. Mean follow-up time was 5.3 years with 17% of patients reaching a follow-up time of over 10 years. There were 47 deaths (25.7%) within the follow-up period. MVR patients were more likely to have higher logistic EuroSCORE, active IE and were less likely to have elective surgery. In multivariate Cox proportional hazards analysis, there was no significant difference in long-term mortality between MVr and MVR groups (hazard ratio 1.09, 95% confidence interval [0.59–2.00]). In Kaplan–Meier analysis, MVR patients had a higher all-cause mortality although there was no significant difference at the endpoint. Propensity score matching analysis showed a significantly higher mortality in the replacement group instead (*p* = 0.002), Subgroup analysis revealed there remained no significant difference in mortality even in patients with active IE (*P*-interaction = 0.859) or non-elective surgery (*P*-interaction = 0.122). MV reoperation (odds ratio 1.00 [0.24–4.12]), post-operative intensive care stay (*p* = 0.9650) and total hospital stay (*p* = 0.9144) were comparable.

**Conclusions:**

Our data demonstrates repair was at least non-inferior to replacement in IE, supporting more aggressive use of repair. There is no reason the general principle of why repair is superior to replacement should not hold in IE, with enough operator expertise. Other experienced units should be encouraged to increase repair rates as feasible in line with current guidelines.

**Supplementary Information:**

The online version contains supplementary material available at 10.1186/s13019-022-01997-2.

## Introduction

Mitral valve repair (MVr) is unquestionably superior to mitral valve replacement (MVR) in non-infective settings due to better preservation of left ventricular function, lower mortality and not necessitating life-long anticoagulation with mechanical valves [1]. We apply this principle into the infective setting, where there remains some controversy for the optimum surgical approach.

Surgical management for native mitral valve infective endocarditis (IE) is indicated in severe mitral regurgitation or obstruction causing refractory pulmonary oedema or cardiogenic shock, in uncontrolled infection, fungal or multi-resistant infections, or in persistent vegetations > 10 mm for prevention of embolism [2]. Even in the infective setting, both the European Society of Cardiology (ESC) and American Association for Thoracic Surgery guidelines recommend repair over replacement where possible, with the aim of total removal of the infected tissue and reconstruction of cardiac morphology [2, 3]. Notably, guidelines suggest extensive destruction of a single leaflet or an abscess does not necessarily preclude valve repair [4].

A recent meta-analysis demonstrated significant survival benefit for MVr over MVR in native MV endocarditis. MVr was also found to be durable and resistant to reinfection [5]. Furthermore, bioprosthetic valves have poor durability with the risk of structural valve deterioration in younger patients, and mechanical valves necessitates lifetime anticoagulation due to the risk of thromboembolic events [6]. However, MVr can be technically challenging due to infected and friable tissue. Nevertheless, studies have reported the feasibility of MVr in up to over 80% of patients in specialised centres [7, 8].

Despite these clear recommendations, MVr rates have lagged. The National Inpatient Sample in the USA report a repair rate of 25%[9] and the Taiwan National Health Insurance program report a repair rate of 21% [10]. This could be due to concerns over the durability of MVr and recurrence of IE especially in active endocarditis and in non-elective surgery. [11, 12]

As such, we aim to detail our experience at a centre with high repair rates[13], describe characteristics of patients who were deemed unsuitable for repair, and describe the long-term outcomes of MVr and MVR in both active and healed native mitral endocarditis. Although in all cases where a durable repair was thought to be feasible as per operator experience it was attempted, we conduct additional analysis comparing MVr and MVR in patients in which repair would theoretically be feasible as per ESC guidelines as retrospectively assessed by operative reports.

## Patients and methods

### Patients

From October 2005 to March 2021, a consecutive series of 211 patients underwent surgical treatment for MV infective endocarditis at our institution. 28 patients with prosthetic valve endocarditis were excluded from our study. A final 183 patients with either active or healed native valve endocarditis were included in our study, of which 85 (46.4%) underwent MVr and 98 (53.6%) underwent MVR. Patients with native, multi-valve disease were included. Comprehensive preoperative, perioperative, and postoperative data were collected retrospectively from our registry. As the study was retrospective and observational, we have local ethics approval for using anonymised patient data for research as per local policy.

The primary outcome was long-term mortality. Long-term follow-up outcome data was obtained from the Office for National Statistics which collects and securely links information about all deaths registered in England and Wales to the Electronic Patient Records at our institution. This allowed for near complete follow-up mortality data (98.9%). Two patients in the replacement group were lost to follow-up after moving abroad. Follow-up was complete in the repair group. Patient status was last confirmed 31 March 2021. Secondary outcomes were any clinically significant post-operative MR (defined as moderate to severe), MV reoperation until last follow-up, reason for reoperation, thromboembolic or haemorrhagic complications including any stroke, and pulmonary, splenic, or renal infarctions, and length of post-operative intensive care unit (ICU) and total hospital stay.

All patients were diagnosed of infective endocarditis based on the modified Duke’s criteria [14] as well as preoperative and intraoperative echocardiography assessed by a cardiologist, and microbiology and pathology confirmation. Active infective endocarditis was defined as patients receiving antibiotics for bacterial endocarditis as per modified Duke’s criteria. Healed infective endocarditis was defined as patients no longer receiving antibiotics for bacterial endocarditis as per modified Duke’s criteria. All patients were treated with antibiotics as per local policy. Most patients received triple therapy comprising of amoxicillin, flucloxacillin, and gentamicin, until sensitivities were obtained. Penicillin allergic patients received vancomycin and gentamicin. If patients had indwelling lines or implanted cardiac devices, they received vancomycin, gentamicin, and rifampicin.

Operative urgency was defined as per Society of Thoracic Surgery data specifications [15]. In brief, salvage surgery was defined as requiring perioperative CPR; emergency surgery was defined as either ischaemic dysfunction or mechanical dysfunction with shock with or without circulatory support; urgent surgery was defined as requiring same day hospitalisation to prevent further deterioration; elective surgery was defined as surgery which can be deferred without increased risk of compromised cardiac output..

For additional analysis, theoretical feasibility of repair for those who underwent replacement were assessed retrospectively based on operative reports by a senior author. As defined by to the ESC guidelines [2], perforations in a single valve cusp or leaflet, multiple ruptured chordae, extensive destruction of a single leaflet and the presence of an abscess do not preclude repair, and could be classed as theoretically feasible for repair. Contraindications to repair included but were not limited to extensive destruction of both leaflets, severe leaflet or annular calcification, severe asymmetric tethering, severe annular dilatation, and access difficulties.

### Surgical procedures

Indication for surgery in all patients were due to failure of or contraindication to conservative management, including haemodynamic compromise, uncontrolled sepsis, antibiotic-resistant infections, abscess, large vegetation, or high risk of embolization. Most surgical approaches were by median sternotomy, with two patients undergoing minimally invasive endoscopic surgery via a right mini thoracotomy. All patients who underwent MVr had an annuloplasty ring, of which 74.1% (n = 60) was the Cosgrove Edwards annuloplasty ring. Majority of patients underwent -plasty procedures, most commonly triangular resection. The remaining 25.9% (n = 21) underwent a patch repair, of which majority were autologous (n = 8), followed by porcine (CorMatrix, ProxiCor, n = 7), bovine (XenoSure, n = 4), equine (n = 1), and unspecified (n = 1). 23.5% of repairs (n = 19) also had artificial chords implanted. In MVR, 81.2% (n = 78) of implants were biological prostheses and 18.8% (n = 18) were mechanical. Selection of prostheses was based on age, operator, and patient preference. Of the MVR group, 70.4% (n = 69) of cases preserved both the anterior and posterior leaflet.

### Statistical analysis

Continuous data are presented as median and interquartile ranges and categorical data are presented as count and percentages. As informed by the Shapiro–Wilk test for normality, all continuous data were non-parametric. Inter-group comparison was based on the Mann–Whitney U test for continuous data and the chi-square or Fisher’s exact test for categorical data as appropriate. Cox proportional hazards regression analysis was performed to assess the relationship between MVr versus MVR and all-cause mortality. Selection of covariates for multivariate analysis was performed based on inter-group differences in baseline characteristics to mitigate the effects of confounding. Model 1 refers to univariate analysis, Model 2 adjusted for urgency and active infective endocarditis and Model 3 adjusted for logistic EuroSCORE to avoid collinearity. To test the robustness of our findings and to identify if specific patient populations would benefit more from either repair or replacement, we carried out subgroup analysis and *P*-interaction tests. Differences in survival rates between groups were estimated using Kaplan–Meier survival curve analysis and the log-rank test. The proportional-hazards assumption was tested. 1:1 propensity score matching to estimate average treatment effects of the treated population was performed to ensure comparability of cohorts. Reoperation rates were analysed by multivariate logistic regression. Other secondary outcomes (e.g., post-operative stay) were assessed using multivariate analysis of covariance or Fisher’s exact test as appropriate. Supplementary analysis was performed similarly with Cox’s hazards model to identify other predictors of mortality. A *p *value below 0.05 in univariate analysis was required for retention in the final multivariate model. All analyses were performed using Stata version 14.1 software.

## Results

The median patient age was 58 years (44–69) and 33.9% were female. Median logistic EuroSCORE was 11.13 (5.32–25.9). 58.5% (n = 107) of surgeries were urgent, 24.0% (n = 44) were emergent, 16.9% (n = 31) were elective and 0.6% (n = 1) was salvage. Table 1 compares the baseline and procedural characteristics of patients by repair or replacement. Patients who underwent MVR had significantly higher logistic EuroSCORE, were less likely to have undergone elective surgery and were more likely to have active infective endocarditis. All other factors including age, gender, history of previous cardiac surgery, dialysis, pulmonary and neurological disease, left ventricular ejection fraction (LVEF), concomitant cardiac and valve surgery, cumulative cross-clamp and bypass time were not significantly different.

**Table 1:** Baseline and procedural characteristics of study participants by repair and replace

	MVr (n = 85)	MVR (n = 98)	*P value
Age (years)	56 (42–70)	60 (46–69)	0.5756
Gender, females	28 (32.9)	34 (34.7)	0.803
BMI	24 (21–27)	23 (21–27)	0.7034
CCS class 3–4 angina	5 (5.9)	3 (3.1)	0.475
NYHA ≥ 3	30 (35.3)	42 (42.9)	0.296
MI within 90 days of surgery	2 (2.4)	0 (0.0)	0.214
Previous cardiac surgery	7 (8.2)	9 (9.2)	0.821
Diabetes	7 (8.2)	13 (13.3)	0.277
Hypertension	35 (41.2)	41 (42.3)	0.935
Current smoker	17 (20.0)	24 (24.5)	0.468
History of dialysis	7 (8.6)	9 (9.8)	0.796
History of respiratory disease	6 (7.1)	16 (16.3)	0.055
History of stroke/TIA	14 (16.5)	25 (25.5)	0.136
Extracardiac arteriopathy	2 (2.4)	6 (6.2)	0.287
Non-SR on admission	9 (10.6)	11 (11.2)	0.891
LVEF			
> 50%	64 (75.3)	66 (67.4)	0.388
31–50%	20 (23.5)	28 (28.6)	
≤ 30%	1 (1.2)	4 (4.1)	
Logistic EuroSCORE	8 (4–18)	15 (6–29)	**0.0007**
Urgency			
Elective	23 (27.1)	8 (8.2)	**0.002**
Urgent	45 (52.9)	62 (63.3)	
Emergency	16 (18.8)	28 (28.6)	
Salvage	1 (1.2)	0 (0)	
Concomitant cardiac surgery			
Valve alone	63 (74.1)	80 (81.6)	0.329
Valve + CABG	8 (9.4)	4 (4.1)	
Valve + other**^**	13 (15.3)	11 (11.2)	
Valve + CABG + other**^**	1 (1.2)	3 (3.1)	
Active IE	51 (60.0)	84 (85.7)	** < 0.0001**
MV Regurgitation	84 (98.8)	92 (94.9)	0.374
Concomitant valve surgery			
Mitral only	71 (83.5)	72 (73.5)	0.205
Mitral + Aortic	12 (14.1)	19 (19.4)	
Mitral + Tricuspid	2 (2.4)	3 (3.1)	
Mitral + Aortic + Tricuspid	0 (0)	4 (4.1)	
Cumulative cross clamp time	76 (55–98)	82 (64–106)	0.1476
Cumulative bypass time	103 (75–123)	111 (85–145)	0.0561
Later year of surgery (early 2005–2012 vs late 2013–2021)	53 (62.4)	62 (63.3)	0.899

Data presented are median (IQR) or number (percentage), where appropriate

Statistical significance (*p* < 0.05) was denoted by bolded values

*MVr* mitral valve repair, *MVR* mitral valve replacement, *CCS* Canadian Cardiovascular Society, *NYHA* New York Heart Association, *MI* myocardial infarction, *TIA* transient ischaemic attack, *SR* sinus rhythm, *LVEF* left ventricular ejection fraction, *CABG* coronary artery bypass graft, *IE* infective endocarditis, *MV* mitral valve

^*^P value was based on chi-square, Fisher’s exact test or Mann–Whitney U test where appropriate

^Other cardiac procedures in descending order of frequency were ASD closures, VSD closures, LA appendage occlusion, and pericardiectomies

Mean follow-up was 64 months (5.3 years) and a maximum of 15.4 years. There were 47 deaths (25.7%) within the follow-up period. There was a higher percentage of long-term all-cause mortality in the replacement group (28.1%) as compared to the repair group (23.5%). There was a higher risk of mortality in MVR than MVr in univariate analysis (hazard ratio, 1.23) although the association was not significant (Table 2). After adjusting for all between-group differences in baseline characteristics including operative urgency, active infective endocarditis and logistic EuroSCORE in Model 2 and Model 3, there remained no difference in all-cause mortality in repair versus replacement, suggesting outcomes from both MVr and MVR could be equivalent.

**Table 2:** Association between repair vs replacement and all-cause mortality

	N	No. of events (%)	Model 1	Model 2	Model 3
			HR (95% CI)	HR (95% CI)	HR (95% CI)
MVr	85	20 (23.5)	Reference	Reference	Reference
MVR	98	27 (28.1)	1.23 (0.69–2.19)	1.09 (0.59–2.00)	0.83 (0.45–1.54)
MVR, but theoretically feasible for repair	33	12 (36.4)	1.56 (0.76–3.20)	1.69 (0.74–3.86)	1.02 (0.47–2.23)

Model 1: univariate analysis; Model 2: urgency and active endocarditis adjusted only; Model 3: logistic EuroSCORE adjusted only

*MVr* mitral valve repair, *MVR* mitral valve replacement, *IE* infective endocarditis, *MV* mitral valve

To evaluate the differences in outcomes for patients in which MVr was theoretically feasible as retrospectively assessed, but MVR was performed due to various reasons such as operator preferences and experience, additional analysis in this patient population was carried out (Table 2). Of the 98 patients who underwent MVR, 33 patients (35.1%) were assessed to have been theoretically feasible for repair as defined by ESC guidelines. Four operative reports were either unavailable or did not contain sufficient information to make a judgement; the rest were unsuitable for MVr. Replacement patients who were technically feasible for repair were significantly less likely to have underwent previous cardiac surgery (*p* = 0.027, data not shown) when compared against patients who were theoretically not feasible for repair (i.e. the remaining of the replacement group). All other demographic variables were comparable. There was a slightly higher percentage of long-term mortality in the replacement population thought to have been feasible for repair (36.4%) compared with all patients who underwent MVR (28.1%). Similarly, even though the HR was higher in this smaller population compared with all MVR patients, all-cause mortality remained comparable with the repair group after adjustment in both Models 2 and 3.

**Fig. 1:**
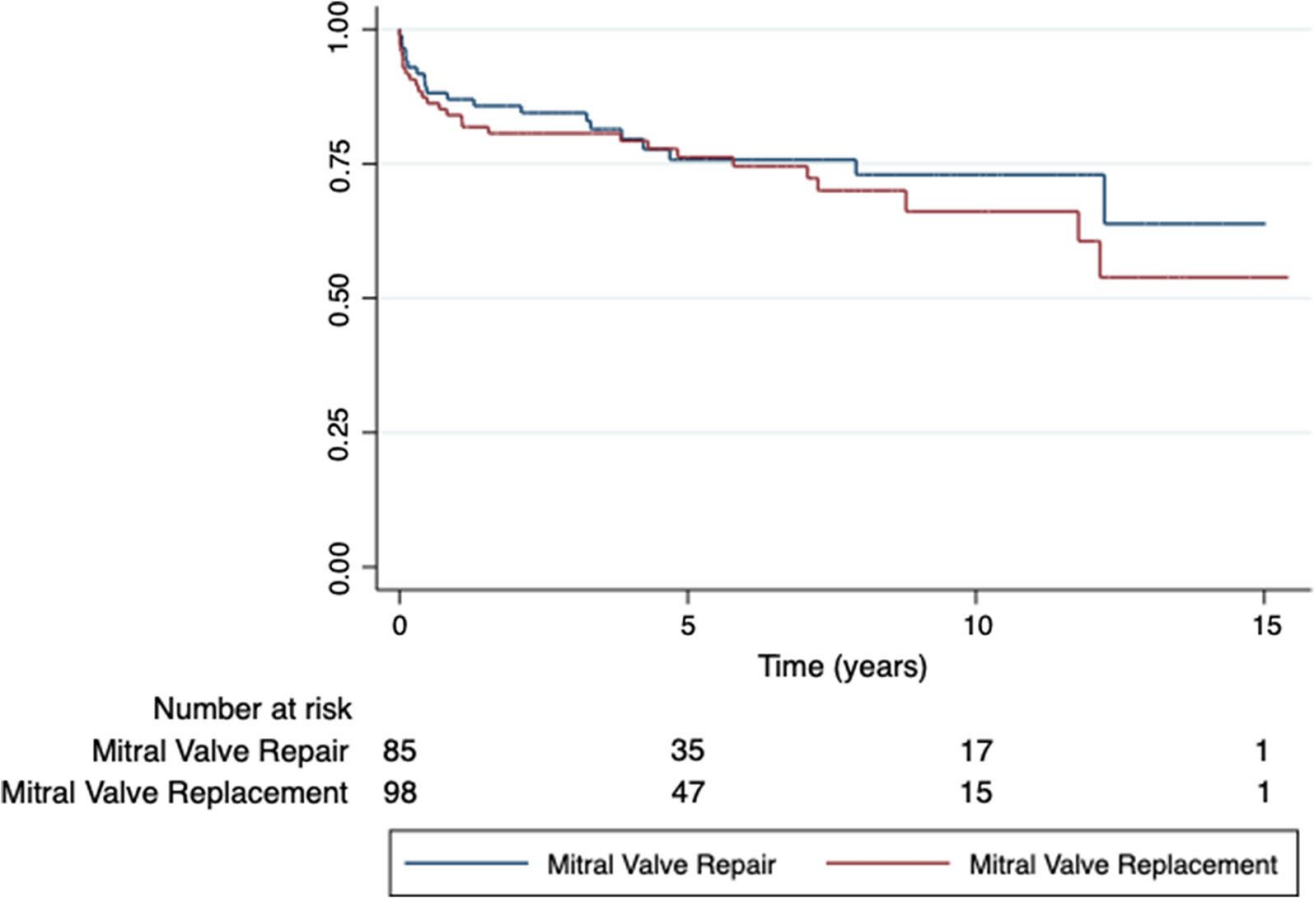
Kaplan–Meier survival curves for mitral valve repair (MVr) vs replacement (MVR). Endocarditis patients with MVR had a greater all-cause mortality than those with MVr. However, there was no significant difference in long-term mortality (*p* = 0.4869). Test of proportional-hazard’s assumption satisfied (*p* = 0.7665). Mean follow-up was 5.3 years and a maximum of 15.4 years

Inter-group Kaplan–Meier survival curve analysis (Fig. 1) showed that although patients who underwent MVR had higher long-term all-cause mortality than those who underwent MVr. However, there was no significant difference at the endpoint (*p* = 0.4869). Test of proportional-hazard’s assumption was satisfied (*p* = 0.7665).

Propensity score matching for all baseline demographics in Table 1 was carried out to ensure all differences between cohorts were captured (Table 3). Nearest neighbour matching was carried out, with a total of 165 patients included, comprising 80 repair and 85 replacement patients. After matching, there were no longer any significant differences in all baseline demographics (data not shown), including Logistic EuroSCORE (which was not initially a matched variable to avoid the effects of collinearity). This reported a significantly higher mortality in replacement patients than in repair patients (*p* = 0.002).

**Table 3:** Propensity matched comparison of all-cause mortality in replacement versus repair

	Coefficient	95% CI	*P* value
Replacement versus repair*	0.1176	0.04–0.19	**0.002**

Statistical significance (*p* < 0.05) was denoted by bolded value

^*^Matched for all baseline demographic characteristics in Table 1 except Logistic EuroSCORE to avoid the effects of collinearity, including age, gender, BMI, CCS status, NYHA status, previous cardiac surgery, diabetes, hypertension, current smoker, history of dialysis, respiratory disease, stroke/TIA, extra cardiac arteriopathy, non-sinus rhythm on admission, LVEF, urgency, and active endocarditis

To evaluate if any subgroup of patients might benefit more from repair or replacement, we conducted subgroup analysis (Table 4) by active endocarditis status at time of surgery and operative urgency. There was a higher percentage of deaths in MVR as compared to repair in the subgroups: healed endocarditis (30.8% compared to 23.5%), active endocarditis (27.7% compared to 23.5%) and in elective surgery (25.0% compared to 4.4%). In urgent, emergency or salvage surgery, the percentage of deaths in repair versus replacement were similar (30.7% compared to 28.4%). However, there were no significant differences in univariate and multivariate analysis with healed or active endocarditis (*P*-interaction = 0.859 or operative urgency (*P*-interaction = 0.122), suggesting potentially comparable results between repair and replacement across these groups although our sample size may be too small to detect significant differences if one exists.

**Table 4:** Association between repair vs replacement and mortality within subgroups

	N	No. of events (%)	Model 1	Model 2	Model 3
			HR (95% CI)	HR (95% CI)	HR (95% CI)
Healed Endocarditis					
MVr	34	8 (23.5)	Reference	Reference	Reference
MVR	14	4 (30.8)	1.47 (0.44–4.89)	0.75 (0.19–2.89)	1.62 (0.46–5.73)
Active Endocarditis					
MVr	51	12 (23.5)	Reference	Reference	Reference
MVR	84	23 (27.7)	1.10 (0.54–2.21)	1.09 (0.54–2.20)	0.85 (0.41–1.77)
Elective					
MVr	23	1 (4.4)	Reference	Reference	Reference
MVR	8	2 (25.0)	6.72 (0.61–74.48)	6.70 (0.61–74.09)	6.80 (0.58–79.25)
Non-Elective					
MVr	62	19 (30.7)	Reference	Reference	Reference
MVR	90	25 (28.4)	0.84 (0.46–1.53)	0.90 (0.48–1.68)	0.63 (0.34–1.18)

Model 1: univariate analysis; Model 2: urgency adjusted in endocarditis subgroups, active endocarditis adjusted in urgency subgroups; Model 3: logistic EuroSCORE adjusted only

*MVr* mitral valve repair, *MVR* mitral valve replacement, *IE* infective endocarditis

*P*-interaction for endocarditis = 0.859

*P*-interaction for urgency = 0.122

Table 5 demonstrates reoperation rates were higher in those with MVR even after adjustment for logistic EuroSCORE, although the results were insignificant (OR 1.12; 95% CI 0.28–4.43). Recurrence of endocarditis as an indication for reoperation was markedly higher in the MVR group at 80% (n = 4 of 5), compared with 25% (n = 1 of 4) in the MVr group, although again insignificant due to low numbers (*p* = 0.206). Remaining indications were valve failure. Additional file 1: Table 1 reports all bacteriological agents involved, split by MVr and MVR. Notably, we find that even the particularly virulent *staphylococcus aureus* was repairable, split evenly between MVr and MVR groups (17.1% versus 20.4%). The one organism in MVr necessitating reoperation for recurrence of infection was aspergillus. Correspondingly in MVR, two cases of reinfection with *Viridans Streptococci* and one each of Enterococcus and from the HACEK group necessitated reoperation.

**Table 5:** Reintervention rates in study participants by MV repair and replacement

	N	No. of events (%)	Model 1	Model 2	Model 3
			OR (95% CI)	OR (95% CI)	OR (95% CI)
MVr	85	4 (4.71)	Reference	Reference	Reference
MVR	98	5 (5.38)	1.09 (0.28–4.19)	1.00 (0.24–4.12)	1.12 (0.28–4.43)

Model 1: univariate analysis; Model 2: urgency and active endocarditis adjusted only; Model 3: logistic EuroSCORE adjusted only

*MVr* mitral valve repair, *MVR* mitral valve replacement, *IE* infective endocarditis

Post-operative ICU stay and total hospital stay were also comparable between repair and replacement groups (Table 6). There was a lower rate of thromboembolic or haemorrhagic complications in MVr patients (5.88% compared with 7.14%), although this was not statistically significant. There were no patients with clinically significant post-operative MR in either group.

**Table 6:** Other secondary outcomes by MV repair and replacement

	MVr (n = 85)	MVR (n = 98)	*P value
Post-operative stay, days	14 (7–33)	20 (11–37)	0.9650
Total hospital stay, days	18 (10–42)	30 (15–47)	0.9144
Thromboembolic or haemorrhagic events	5 (5.88)	7 (7.14)	0.548

Data presented are median (IQR)) or number (percentage) where appropriate

*MVr* mitral valve repair, *MVR* mitral valve replacement

^*^P value was based on ANCOVA, adjusted for Logistic EuroSCORE, or Fisher’s exact test where appropriate

Supplementary analysis (Additional file 1: Table 2) to identify other risk factors for mortality revealed that in multivariate analysis, myocardial infarction within 90 days of surgery (HR 10.57 [1.41–79.08]), history of dialysis (HR 7.89 [3.35–18.58]) and LVEF (HR 2.33 [1.24–4.38]) were significant predictors for mortality. However, the low numbers of patients with the former two characteristics led to the wide confidence intervals.

## Discussion

The current study presents a 15-year experience in a high-volume single-centre comparing the long-term outcomes of MVr and MVR in native valve endocarditis. As a specialist centre, we found that MVr was feasible in 46% of patients, higher than numerous contemporary studies [9, 10, 16, 17]. Patients unsuitable for MVr were more likely to be higher risk (higher logistic EuroSCORE, non-elective, active IE). After multivariate adjustment, we found MVr to be non-inferior to MVR in both active and healed native MV endocarditis. However, in propensity score matching that we found a significant difference in mortality. This suggests after accounting for all differences in baseline factors, MVr is superior to MVR, even in an infective setting.

The main advantages of repair are the improved preservation of left ventricular function through the preserved continuity of the mitral apparatus [18], lower operative risks [19], and through freedom from complications related to prosthetic valve implants including risk of thromboembolism, lifetime anticoagulation and subsequent risk of haemorrhage, structural valve deterioration and prosthetic-valve endocarditis, especially in an active infection [1, 20, 21]. However, depending on the extent of valve destruction on presentation and the difficulty of operating on friable, actively infected tissue, some patients might not be suitable for repair. We attempted to mitigate this by retrospectively assessing operative reports to compare a subset of patients who were theoretically feasible for repair.

Although long-term mortality was lower in patients who underwent MVr, we did not report a statistically significant difference in HR. This is well corroborated in previous retrospective observational studies which found that despite MVR patients being sicker, outcomes between MVr and MVR remained comparable [17, 22–25]. The lack of statistical difference in long-term mortality could be due to the low power of these individual studies including the current study. Numerous other studies reported that MVr conferred a significant survival benefit over MVR, including a recent meta-analyses [5] and notably, the two largest multi-centre studies, Gammie et al. on 6627 patients in the USA[26] and Lee et al. on 704 patients in Taiwan [10]. This could be due to increased power with larger sample sizes. We only report significantly higher mortality in MVR when propensity score matching was carried out, potentially due to capturing differences in baseline demographics previously not accounted for in the Cox model despite multivariate adjustment for significant differences in baseline characteristics. Differences could also be due to variation in surgical techniques. Over 70% of our MVRs conserved both the anterior and posterior subvalvular apparatus which would preserve the continuity between the mitral annulus and left ventricular wall, aiding preservation of left ventricular function [18], similar to in MVr.

We report comparable MV reoperation rates, recurrence of infection necessitating reoperation (although markedly higher in MVR patients, 80% vs 25%, this was not statistically significant), and thromboembolic or haemorrhagic complications in both groups. Ruttman et al. report MVr patients had significantly lower incidence of MV reoperation and recurrence of endocarditis [27]. Lee et al. also report MVr patients were significantly less likely to have MV reoperation, any stroke, or major bleeding [10]. Previous studies have investigated other measures of event-free survival. Wilhelm et al. found MVr patients had a significantly lower incidence of atrial fibrillation (reducing the need for anticoagulation and thus the risk of haemorrhage) as well as the need for pacemaker implantation, even though there was no significant difference in long-term mortality [25].

In subgroup analysis, we found that outcomes in MVr and MVR were comparable even in active IE and in non-elective surgery. Lee et al. found that MVr was associated with a lower rate of mortality in active IE, but not in emergent surgery [10]. In contrast, Muehrcke et al. reported that while MVr was associated with lower mortality in both active and healed IE, in the active IE subgroup, the results were not statistically significant [28].

Comparison between studies is difficult, given the variety of definitions of active IE, non-elective surgery, and surgical techniques. Furthermore, peri-operative management and timing of surgery is arguably more variable between centres in IE in comparison to other cardiac surgery presentations (particularly when including historical data) and therefore attributing outcomes singularly to surgical strategy may be flawed.

The strengths of this study include our high repair rate, long-term follow-up and comprehensive peri-operative data allowing for multivariate adjustment. Complications discussed were thorough including embolic events, long-term MV reoperation with corresponding indication such as recurrence of endocarditis or valve failure, although data regarding heart failure were unavailable. The limitations of our study include limitations inherent to all studies of retrospective, observational and non-randomised design. However, randomised controlled trials on this topic are not feasible due to the degree of inter-patient variability in MV destruction and ethical issues given guidelines clearly recommending MVr over MVR when possible. Although selection of procedure may be affected by operator bias, depending on preference or seniority of the surgeon and the learning curve attained, we retrospectively reviewed operative reports to further clarify the relationship in the population of MVR patients for whom repair could theoretically be feasible. However, this evaluation was both retrospective and subjective; some of these patients could have still been unsuitable for repair due to reasons unmentioned in the operative reports, such as access difficulties. While it is possible that there is confounding due to differences in baseline characteristics between the groups, we believe that the effect of confounding is unlikely to be extensive due to multivariate adjustment of important variables and propensity matching analysis to ensure matched cohorts for comparison. The study also has no morphological or echocardiography data which would have been instructive on the pathological substrate of the patients. Destruction extent score and repair complexity score which would have shed light on choice of procedure were also unavailable. Data for intravenous drug use, both a risk factor for severity of leaflet destruction on presentation and subsequent mortality, were unavailable. As a specialist high repair rate centre, our results might not be generalisable across other centres with less experience in MVr in IE. Lastly, future multi-centre studies with larger sample sizes and comprehensive follow-up data are needed to confirm our findings.

## Conclusion

This study presents the largest known single-centre experience in the UK to date and has long-term data up to 15 years demonstrating at least non-inferiority of repair to replacement in the context of MV IE with excellent results. There is no reason the general principle of why repair is superior to replacement should not hold even in the infective setting, with enough careful patient selection and operator expertise. These data support more aggressive use of repair in MV IE and should encourage other experienced units to increase repair rates as feasible in line with current guidelines.

## Supplementary Information

**Additional file 1**. **Supplementary Table 1.** Bacteriological agents involved by MVr and MVR. **Supplementary Table 2.** Association between other risk factors and mortality.

## Data Availability

Available upon request from authors.

## Abbreviations

MVr: Mitral valve repair
MVR: Mitral valve replacement
IE: Infective endocarditis
MV: Mitral valve
ICU: Intensive care unit
LVEF: Left ventricular ejection fraction

## Declarations

### Previously presented at meeting

Society of Cardiothoracic Surgeons Annual Meeting, Belfast, 8-10th May 2022.
