# Supplementary material for: Should more patients be offered repair for mitral valve endocarditis? a single-centre 15-year experience
Source: J Cardiothorac Surg. 2022 Sep 30;17:243. doi: 10.1186/s13019-022-01997-2 (PMC9523968; doi:10.1186/s13019-022-01997-2)
Supplement: Supplementary file 1 — Additional file 1. Supplementary Table 1. Bacteriological agents involved by MVr and MVR. Supplementary Table 2. Association between other risk factors and mortality. [file 13019_2022_1997_MOESM1_ESM.docx]

| **Supplementary Table 1. Bacteriological agents involved by MVr and MVR** | | |
| --- | --- | --- |
|  | **MVr**  (n=85) | **MVR**  (n=98) |
| *Staphylococcus aureus* | 12 (17.1) | 19 (20.4) |
| Coagulase negative *Staphylococcus* | 1 (1.43) | 6 (6.45) |
| *Viridans Streptococcus* | 10 (14.3) | 26 (28.0) |
| Group A *Streptococcus* | 2 (2.86) | 0 (0.00) |
| Group B *Streptococcus* | 1 (1.43) | 4 (4.30) |
| *Mutans* *Streptococcus* | 2 (2.86) | 2 (2.15) |
| *Enterococcus* | 6 (8.57) | 6 (6.45) |
| HACEK group | 5 (7.14) | 3 (3.23) |
| Fungal | 1 (1.43) | 0 (0.00) |
| Other *Streptococci^a^* | 7 (10.0) | 13 (14.0) |
| Other *Staphylococci^b^* | 1 (1.43) | 3 (3.23) |
| Others^c^ | 8 (11.4) | 5 (5.38) |
| Negative* | 14 (20.0) | 6 (6.45) |
| Abbreviations: HACEK, Haemophilus, Aggregatibacter, Cardiobacterium, Eikenella, Kingella; MVr, mitral valve repair; MVR, mitral valve replacement.  ^a^Other *Streptococci* include, in descending order*, Streptococcus bovis, Streptococcus pneumoniae*, unidentified *Streptococcus*, *Streptococcus canis*, and *Streptococcus dysgalactiae*.  ^b^Other *Staphylococci* include, in descending order*, Staphylococcus warneri* and *Staphylococcus pasteuri*. | | |

| **Supplementary Table 2. Association between other risk factors and mortality** | | | | |
| --- | --- | --- | --- | --- |
|  | **Univariate** | | **Multivariate** | |
|  | **HR (95% CI)** | **P-value** | **HR (95% CI)** | **P-value** |
| Age | 1.02 (1.00-1.04) | **0.035** | 1.01 (0.98-1.04) | 0.432 |
| Gender, females | 0.93 (0.50-1.73) | 0.818 |  |  |
| BMI | 1.00 (0.98-1.01) | 0.691 |  |  |
| CCS class 3-4 angina | 3.82 (1.34-10.90) | **0.012** | 0.13 (0.01-1.06) | 0.057 |
| NYHA≥3 | 1.53 (0.86-2.71) | 0.149 |  |  |
| MI within 90 days of surgery | 8.77 (2.05-37.56) | **0.003** | 10.57 (1.41-79.08) | **0.022** |
| Previous cardiac surgery | 1.48 (0.59-3.75) | 0.407 |  |  |
| Diabetes | 1.80 (0.80-4.03) | 0.154 |  |  |
| Hypertension | 2.20 (1.23-3.93) | **0.008** | 1.09 (0.52-2.30) | 0.822 |
| Current smoker | 1.71 (0.92-3.15) | 0.088 |  |  |
| History of dialysis | 7.48 (3.65-15.32) | **<0.001** | 7.89 (3.35-18.58) | **<0.001** |
| History of respiratory disease | 2.64 (1.34-5.19) | **0.005** | 1.46 (0.58-3.69) | 0.420 |
| History of stroke/TIA | 0.95 (0.46-1.98) | 0.898 |  |  |
| Extracardiac arteriopathy | 9.66 (3.62-25.75) | **<0.001** | 2.91 (0.81-10.51) | 0.103 |
| Non-SR on admission | 1.49 (0.67-3.33) | 0.330 |  |  |
| LVEF | 2.65 (1.61-4.36) | **<0.001** | 2.33 (1.24-4.38) | **0.009** |
| Logistic EuroSCORE | 1.03 (1.02-1.04) | **<0.001*** |  |  |
| Urgency | 1.79 (1.16-2.75) | **0.008** | 1.28 (0.77-2.14) | 0.345 |
| MVr vs MVR | 1.23 (0.69-2.19) | 0.488 |  |  |
| Concomitant cardiac surgery | 1.47 (1.08-1.99) | **0.013** | 1.44 (0.95-2.18) | 0.082 |
| Concomitant valve surgery | 1.13 (0.75-1.71) | 0.551 |  |  |
| Active IE | 1.27 (0.65-2.48) | 0.478 |  |  |
| MV Regurgitation | 0.87 (0.31-2.45) | 0.790 |  |  |
| Cumulative cross clamp time | 1.01 (1.00-1.01) | **0.009** | 1.01 (0.99-1.04) | 0.219 |
| Cumulative bypass time | 1.01 (1.00-1.01) | **0.010** | 0.99 (0.98-1.01) | 0.576 |
| Time of surgery (early 2005-2012 vs late 2013-2021) | 0.85 (0.46-1.57) | 0.598 |  |  |

Abbreviations: BMI, body mass index; CCS, Canadian Cardiovascular Society; NYHA, New York Heart Association; MI, myocardial infarction; TIA, transient ischaemic attack; SR, sinus rhythm; LVEF, left ventricular ejection fraction; CABG, coronary artery bypass graft; IE, infective endocarditis; MV, mitral valve.

*Omitted in multivariable model to avoid collinearity
